# Citizens’ Communication Needs and Attitudes to Risk in a Nuclear Accident Scenario: A Mixed Methods Study

**DOI:** 10.3390/ijerph19137709

**Published:** 2022-06-23

**Authors:** Joel Rasmussen, Mats Eriksson, Johan Martinsson

**Affiliations:** 1 Crisis Communication Centre, School of Humanities, Education and Social Sciences, Örebro University, 701 82 Örebro, Sweden; mats.eriksson@oru.se; 2 Medical Radiation Physics, Lund University, 205 02 Malmö, Sweden; johan.martinsson@med.lu.se

**Keywords:** nuclear accidents, decontamination, risk attitudes, communication preferences, focus group interviews, mixed methods research

## Abstract

The potential devastation that a nuclear accident can cause to public health and the surrounding environment demands robust emergency preparedness. This includes gaining a greater knowledge of citizens’ needs in situations involving radiation risk. The present study examines citizens’ attitudes to a remediation scenario and their information and communication needs, using focus group data (n = 39) and survey data (n = 2291) from Sweden. The focus groups uniquely showed that adults of all ages express health concerns regarding young children, and many also do so regarding domestic animals. Said protective sentiments stem from a worry that even low-dose radiation is a transboundary, lingering health risk. It leads to doubts about living in a decontaminated area, and high demands on fast, continuous communication that in key phases of decontamination affords dialogue. Additionally, the survey results show that less favorable attitudes to the remediation scenario—worry over risk, doubt about decontamination effectiveness, and preferences to move away from a remediation area—are associated with the need for in-person meetings and dialogue. Risk managers should thus prepare for the need for both in-person meetings and frequent information provision tasks, but also that in-person, citizen meetings are likely to feature an over-representation of critical voices, forming very challenging communication tasks.

## 1. Introduction

Major nuclear power plant (NPP) accidents are some of the most challenging disaster scenarios, affecting public health, the environment, and wildlife [1]. They present transboundary challenges in the sense that the risks are difficult to contain spatially and temporally [2]. Such risks necessitate high levels of emergency and disaster preparedness, including not only implementing safety procedures within nuclear power plants [3] and establishing decontamination plans [4] but also ensuring that surrounding communities can act safely if an accident occurs [5]. Despite the severity of larger accidents, international and national governing bodies express high ambitions regarding the ability to recover and restore the affected surroundings. If radiation levels in inhabited areas are deemed possible to reduce to below the health hazard limits, extensive decontamination is likely to be carried out, with the expectation that evacuees will return [4,6].

However, NPP accidents have revealed the challenges in communicating information to worried and distrustful citizens [7,8,9]. In the event of an accident, the difference between “the people and the plan” can be significant [5]. If the living conditions are not tolerated and people prefer to move away permanently, decontamination may become unreasonably expensive per resident [10]. It has therefore been argued that to reach a high degree of acceptance and effectiveness, nuclear emergency planning needs to integrate stakeholder views [11,12]. As accidents affecting communities are rare, knowledge about stakeholder views on risks, as well as safety measures and communication needs, is difficult to attain. While extensive research has been conducted in Japan since the Fukushima Daiichi NPP accident [13], other parts of the world could benefit from more research. This study therefore aims to analyze citizens’ attitudes to both risk and communication needs in an NPP accident and remediation scenario in northern Europe. To develop in-depth and contextualized knowledge, we employ a sequential, mixed methods research design [14] that combines focus group interview results and survey data, thereby adding novel insights to research streams on stakeholder views of radiation risk, decontamination, and risk communication.

Previous research has shown that when citizens are permitted to again live in a decontaminated area, younger adults, women, and parents report particularly high levels of worry and, sometimes, the desire to avoid risk altogether by starting over in an unaffected area [8,10,15,16]. Citizens also judge radiation risk in connection with nuclear accidents as particularly high and unpleasant [17,18,19], as it is ranked at the top among other so-called dread risks [20,21]. As such, the public can be said to perceive greater risk than experts do [22]. Lay sensemaking about risk differs from scientific risk analysis in that it draws more on the precautionary principle and on specific case stories, and less on statistical, group-level risk [23].

Knowledge of actual hazards has also improved over time. Limit values for radiation dose have previously been determined based on the thresholds for an average human being, a median measure of the population. However, more recent studies show that female sex and low age are associated with a higher lifetime attributable risk (LAR) of cancer when exposed to low-dose radiation. Age is particularly significant, as the LAR of cancer, at a 137Cs ground deposition of 1.0 MBq/m^2^, is 5.4% for newborn girls, which is higher than that for 30-year-old women by a factor of 4.5 [24]. Because the limit for evacuation after both the Chernobyl and Fukushima Daiichi NPP accidents was set at a ground deposition of 1.5 MBq/m^2^ [25], there have arguably been objective reasons for having concerns. In addition to the LAR of cancer, adverse effects on mental wellbeing are well documented (e.g., [26,27,28]).

Government agencies have historically considered risk communication as the solution to many of the abovementioned issues and differences in risk perception, and they have attempted to transfer information from experts to ordinary people [22]. Risk communication is considered a crucial and curative part of the overall emergency management cycle of nuclear energy systems, enhancing awareness, understanding, the implementation of safety measures, and ultimately, the return to normal life [29]. Similarly, the provision of risk information during remediation work has been of great importance for increasing public compliance with recommended measures, according to several research studies [30,31,32]. When the Fukushima Global Communication Program was evaluated, the evaluators stated that risk communication played “an integral role in shaping individual risk perception as well as risk aversion or reduction behavior” ([33] p. 2). Yet there is also evidence of government representatives being overconfident regarding the effects of communication, and of conflicts with stakeholders persisting continuously [34,35]. As Slovic [22] showed, disagreements are rooted in the fact that stakeholders’ evaluations of the fundamental issues of risk, responsibility, and communication procedures differ.

The current study assesses stakeholder views as attitudes encompassing important orientations and reactions to risk [36,37]. We assume that attitudes entail “a psychological tendency that is expressed by evaluating a particular entity with some degree of favor or disfavor” ([36] p. 1). One of the more common attitudinal responses to risk is worry, which factors into people’s behavioral responses [38] and communication needs [39,40]. In communities facing a crisis, worry amplifies the importance of social ties [41]. Usually, a person who worries is uncertain about the outcome of a process and has negative expectations. The person is concerned about future events and feels uneasy [42]. When people face a risk, worry represents a relatively irrepressible thought process [43]. Worry, according to Griffin et al. ([39] p. 29), “can affect the amount of attention a person pays to information about a threat and the way he or she interprets ambiguous events”. Studies dealing with decontamination management find a relationship between worry and information needs. In one study [44], during a simulated incident including decontamination, the most stressed respondents asked for more information about the decontamination process. Although existing research emphasizes that higher levels of worry predict greater information needs, we know little about whether a certain mode of communication is needed more, such as meetings with the opportunity for dialogue (vs. written information). Our first research question, therefore, explores both the quantitative and qualitative dimensions of this problem:

*RQ 1: How do respondents express worry over radiation risk and their information and communication needs, and is worry over risks associated with a greater need for either dialogical or monological communication at the group level?*

Attitudes to risk moreover comprise behavioral intentions or, as Rohrmann ([45] p. 4) referred to them, “intentions to evaluate a risk situation in a favorable or unfavorable way and to act accordingly” (emphasis added). Our study therefore also explores intentions to either move away from or stay in the remediation area, with the former naturally implying a less favorable attitude toward the remediation scenario. It thereby measures the extent to which people are inclined to wish to avoid risk and to choose a “path that does not touch on the risk” ([46] p. 122). Griffin, Neuwirth, Dunwoody and Giese [39] demonstrated that higher levels of worry increase the need for information, indicating that those expressing risk-averse preferences in the remediation scenario may also express greater information and communication needs. Still, worry and a lack of control can reach such levels that people instead avoid risk information and the risk itself [47]. Accordingly, there seems to be a lack of knowledge about the preferred modes of communication. The next research question, therefore, is as follows:

*RQ 2: How do respondents express intentions to either accept or avoid living in the remediated area, and are preferences to avoid and even move away from the remediated area associated with a greater need for either dialogical or monological communication at the group level?*

The third dimension of risk attitudes explored in this study is how much respondents believe in the authorities’ safety measures (i.e., that decontamination leads to a sufficiently safe and functioning life). Löfstedt ([48] p. 6) stated that trust is “an expression of confidence between the parties in an exchange transaction”, and that it is vital in crisis management. The level of trust in the information source has been shown to impact the extent to which crisis information is shared with others and, thus, its overall impact [49]. Trust in the source delivering risk information has also been shown to increase the acceptance of risk messages [50]. The reduced effectiveness of risk communication has been explained by citizens’ limited trust in responsible institutions [22,51]. In this respect, there are favorable conditions for crisis management authorities in our study context, since Sweden is described as a high-trust society, with a high degree of gender equality that reduces differences in risk perception [52]. Relating to trust, our third research question focuses on respondents’ belief in the effectiveness of remediation measures and its relation to information and communication preferences:

*RQ 3: How do respondents express their belief, or lack thereof, in remediation measures, and is belief in the effectiveness of remediation measures associated with a greater need for either dialogical or monological communication at the group level?*

Each of the research questions is studied by way of a combination of survey data and focus group interviews, as outlined in the following section.

## 2. Materials and Methods

To analyze citizens’ attitudes to risk and their information preferences in a decontamination scenario—and any associations between these classes of attitudes—this study used a sequential, explanatory, mixed-methods design. In addition to a qualitative focus group study (sub-study 1, n = 39), a survey (sub-study 2, n = 2291), was conducted. Both sub-studies were used to help explain and increase the validity of the results [14,53,54]. As such, this study may help fill a need in the risk and crisis research for mixed-methods studies that yield nuanced and context-sensitive knowledge, which may inform the tasks and decisions of professionals [55]. As no nuclear accident affecting surrounding communities has occurred in or around the Nordic region for decades, an interdisciplinary research group created a realistic scenario (see Appendix A) that was presented to all respondents in the survey and the focus groups.

### 2.1. Sub-Study 1: Focus Group Interviews

Ten groups of citizens were interviewed, with each group consisting of three to six demographically homogeneous participants, to allow the conversations to be as open as possible [56]. In total, 21 men and 18 women were interviewed, from young adults in their 20s to middle-aged citizens and older pensioners, with the average age being 49 years old. We formed groups of participants with varying backgrounds to reflect some of the diversity of Swedish society and to allow a breadth of attitudes on the issues under study, which demographics have been shown to influence [15,16]. Participants were recruited after requests were sent to organizations and associations that could not be assumed to have any position on nuclear or environmental issues; usually, one person gathered acquaintances to form a focus group. Each participant received a movie ticket or gift certificate, at a value of 15.00 USD. The focus groups were designed as follows:

- Group 1: Senior men, six participants
- Group 2: Senior men, four participants
- Group 3: Young women, five participants
- Group 4: Young women, three participants
- Group 5: Senior women, five participants
- Group 6: Senior women, three participants
- Group 7: Middle-aged men, four participants
- Group 8: Middle-aged men, three participants
- Group 9: Young men, four participants
- Group 10: Middle-aged women, three participants

Drawing on Braun and Clarke [57], thematic analysis was used to examine the interviews. This work consisted of identifying themes and sub-themes within the framework of the study’s research questions. Themes consist of empirical material that is characterized by a certain mutual similarity and distinctiveness in relation to the rest of the material. We analyzed statements from respondents in all groups to achieve a breadth of views and maximize the possible lessons learned rather than to make quantitative claims. When all relevant material was coded, we developed both typical and theoretically significant observations. Following Kvale and Brinkmann [58], we used interview analysis techniques consisting of both summarizing material and quoting the respondents’ actual words. The respondents’ statements are referenced in this paper (e.g., R1:2 = respondent number 2 in group 1) to ensure transparency and to show that the analysis draws on a breadth of focus groups and respondents.

### 2.2. Sub-Study 2: The Survey

In this sub-study, a survey was sent out by email as a part of a citizen panel. A total of 3800 adult Swedish citizens were invited to participate in the study, from which 2291 did participate (a maximum of 3 reminders were sent out). The sample was stratified according to gender, age, and education. Sample errors consisted of some instances of missing data. Between 2% and 6% of the units lacked a response to some of the questions that were part of this study and, thus, were excluded. Accordingly, the response count (n) may vary slightly from one question to another. The significance level was set at *p* < 0.05.

In line with the requirement to measure attitude based on at least three variables [59], we examined attitudinal positions concerning decontamination as follows. The first question stated, “Would you feel worried to some extent over radioactive substances in your home, even though measurements show that the radiation levels are harmless?” The second question read, “To what extent do you believe the authorities can restore your home to safe levels through remediation?” The choice of answers given for these questions were as follows: “To a very small extent”, “To a somewhat small extent”, “To a neither small nor large extent”, “To a somewhat large extent”, and “To a very large extent”. Third, we asked, “How likely is it that you would continue to live in your home after it has been declared safe by the authorities?” The multiple-choice answers given were, “Very likely”, “Somewhat likely”, “Not very likely”, and “Not at all likely”.

We then analyzed three variables related to information needs. An overarching question was asked: “When your neighborhood is decontaminated, how often and in what way would you like to get information from the authorities about the clean up during the months (up to a year) when the decontamination is in progress?” The respondents were then asked to give their answer regarding three different media forms—letter or email, telephone, or personal meeting—and regarding their preferred contact frequency—every other week, once a month, every other month, or every six months. To test for any association between attitudes to risk and information needs, we used the Kruskal–Wallis non-parametric test. Results are shown in bar charts, but also in tabular form in Appendix B (Table A1 and Table A2).

## 3. Results

### 3.1. Focus Group Discussions on Radiation Risk and Worry

We will now describe in more detail, based on our focus group interviews, what worry over risk in a remediation scenario entails. To begin with, the interviewees assessed very serious risk, yet this also varied greatly, which was expected given the reports in previous research on perceived “dread risk” [20,26] and given the statistically significant variations according to demographics such as age, gender, and family situation [15]. The notion that high levels of radiation from radioactive material can be fatal, coupled with the fact that the risk is invisible to the eye and difficult to control both spatially and temporally, is something that seems to trigger a great deal of discomfort. “There will be diseases, there will be cancer and all that”, said a male social worker in his 40 s (R7:3). “It might just make me a little extra attentive if you do not see or smell it or anything”, a woman, also in her 40 s, explained (R10:1). Similarly, a female senior citizen asked, “And how far away is it still harmful or not? How would you know?” (R6:3). The perception of high risk was confirmed by the fact that, even among groups that previous research has demonstrated to be the least worried, such as elderly white men [60], participants did indeed express high levels of concern. One of the elderly men stated the following: “If you have ended up in such a position, you are always worried. Even if the authorities say it’s harmless, so How do they know? What will happen in 20 years? Does it affect my body?” (R1:1).

The results that concern the seriousness of the risk and its transboundary characteristics are summarized in Table 1, along with additional themes that concern discussions on risk. Discussions in all our focus groups featured expressions of worry about the risk to the health of either one’s children, grandchildren, or future children. A man in his 20 s expressed a reluctance to see children exposed to possible risk: “within your radius it may be safe, but I still don’t think you want to expose your children to the potential risk of something happening” (R9:2). Naturally, the groups containing retirees pointed out concerns about the safety of grandchildren; an elderly man rhetorically asked, “But do you dare, for example, to have visits from children and grandchildren for a longer stay in the area?” (R1:5).

Another novel finding from the focus groups was the worry some expressed regarding the wellbeing of animals. Such concerns could be expected in an agricultural society but are apparently also voiced in more urban environments, where it is common to have a pet that sometimes needs to be outside. These discussions about the potential risk to animals were initiated partly because the scenario specifies that free movement will not be possible given that the abundance of vegetation surrounding a neighborhood cannot be decontaminated. For example, a middle-aged woman explained that her “cat Emmet could not go into the forest grove, and then we would probably not move back actually”, thus valuing the risk for the family cat as too high (R10:2). Another participant incorporated dogs into the conversation about the risk of radiation: “If you are going out with a dog or similar, they may become contaminated in some way” (R8:3). Respondents thus assessed risk quite skeptically when assuming responsibility for the health of family members and domestic animals.

### 3.2. Focus Group Discussions about Communication Needs in a Remediation Scenario

The following results from the focus groups demonstrate the breadth of the perspectives that informants applied to their communication needs in the scenario. Most groups expressed preferences for quite frequent information as well as face-to-face meetings. Fast information provided via the internet was taken for granted by some; one of the younger women jokingly described—with strong support from other respondents in her group—her high demand for easily accessible, frequent, and even interactive live information in the clean-up scenario: “I want a live stream with a picture of my house and all these measuring devices along the entire edge. I want to know exactly what’s going on all the time. And I would have put up a screen, like this. No, but I would have liked a lot of information, and frequently as well.” (R3:5).

Similarly, a group of men in their 40s mentioned that they would welcome updates obtained on demand through a system of detectors connected “to some app [on their phone] to get today’s values, becquerel or radium, or whatever it is—to see the situation” (R8:3). The interviews thus demonstrated the possibility of drawing on some of the more recent communication technologies in conveying information about the remediation.

The focus groups also show that respondents wanted to know such things as what the remediation plan looks like, possible deviations in the work, and how long the clean-up will take. Opportunities to ask questions about the remediation process and its risks, as well as to meet those responsible for the remediation, were requested above all in the initial stage of the remediation process. In the groups containing younger women, it was believed that such ongoing “listening” and “transparent” communication work during the decontamination process could lead to less worry and increased trust. Table 2 summarizes these and other views regarding the information and communication preferences expressed in the focus groups.

Simultaneously, there was a general concern and doubt expressed about the ability of the responsible actors to even manage to conduct such transparent and dialogical communication activities (e.g., through group or personal meetings). One of the elderly men stated the following: “But the question is whether they can handle it when they are cleaning up. Do you have that organizing ability then? Not in the beginning, you definitely don’t. But of course, it [decontamination] spans a year, by then they must have built something up.” (R2:1). The respondents expressed concerns that authorities could not cope with the clean-up situation and all communication issues simultaneously.

For a few respondents in several groups—regardless of age and gender—the worry and uncertainty surrounding such a scenario were so strong that they believed their attitudes could turn into “outrage” and agitation related to receiving government information. The scenario aroused such strong feelings of uneasiness and levels of worry that some older female respondents believed that life in the area would be a thing of the past in such a scenario: “I do not know if I need it [information], because it’s over. I think so”, stated one woman, with the agreement of the others in her group (R5:4). They saw it as a possibility that they would have little need for information at all and that they would not be influenced or persuaded by frequent monological information or dialogical communication from the authorities.

Although the answers varied among respondents, a recurring interpretation was that, despite reassuring messages from the authorities, one would feel worried. “Even if they say it is safe, it will probably stay in one’s head somehow”, said a woman in her 20s (R3:5), referring to her worry over radiation risk. Another group of women emphasized that they “do indeed trust the authorities” (R10:1) but that their worry was rather rooted in the invisibility and severity of the risk.

### 3.3. Worry over Ionizing Radiation Is Associated with Preferences for More Frequent Dialogical Communication

The analysis of the survey data shows that there is an overall positive relationship between the level of worry over risk and the preference for modes of communication that enable some level of dialogue, such as asking questions or making suggestions. However, we found no similar, clear association between the level of concern about ionizing radiation and the preference regarding written information (cf. Table A2, Appendix B). Thus, the differences shown below concern the use of modes of communication that enable real-time dialogue.

**Figure 1:**
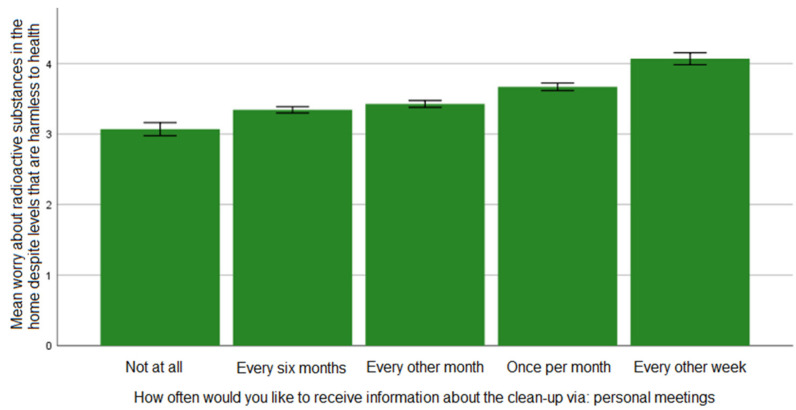
The association between level of worry over ionizing radiation and preference for information about remediation through personal meetings (error bars: ±1 standard error [SE]).

As shown in Figure 1, the results demonstrate a monotonic, almost linear relationship between the studied variables: the greater the expressed concern, the greater the need for frequent information through personal meetings. The analysis showed statistically significant differences between the following variable values: “every other week” and all other values (*p* < 0.05); “once per month” and all other values (*p* < 0.05); “every other month” + “every six months” and all other values (*p* < 0.05); and “not at all” + “every six months” and all other values (*p* < 0.05).

### 3.4. Focus Group Discussions on Decontamination Effectiveness

Another important area of discussion in the focus groups was the challenges associated with remediation, as summarized in Table 3. The view that knowledge of decontamination is lacking reappeared again and again. On the one hand, the uncertainty seemed to justify the application of the precautionary principle—if we do not know exactly how effective remediation is, why take the chance? A man in his 30s said that despite decontamination, “there is always a risk that something is left, so why should I risk myself? And there is probably never anyone who can know 100% for sure” (R8:1). On the other hand, the lack of knowledge and experience regarding decontamination was believed to include those who would be performing the decontamination procedure, because the type of accident is so unusual. It is widely understood that when people do something rarely, as opposed to frequently, they are worse at performing the task. A man in his 40s was skeptical of the effectiveness of remediation precisely because “there is no experience of it except a few isolated cases around the world” (R7:1). A middle-aged woman also emphasized, “I have a high level of trust in the authorities, but cleaning up nuclear waste is not something they practice very much” (R10:2). However, if they were to live in the area after the clean-up, several respondents emphasized the value of reducing the lack of knowledge among citizens by involving them in radiation measurement.

Moreover, in their assessment of the effectiveness of decontamination, respondents drew on their understanding of radiation as posing a transboundary risk, as summarized in Table 3. Some statements emphasized that radioactive material is difficult to contain spatially. Respondents remarked that a contaminated surface can “leak” to other places. When an older group of men were asked if they thought the clean-up effort would be effective, one of them replied that radioactive material “creeps in everywhere, you know. So, I do not think so” (R1:6). Although surfaces can be washed, radioactive material was believed to travel further: “It goes down into the groundwater, you see. No, I don’t believe in it” (R1:6). Likewise, an elderly woman wondered, “Can you really clean everything up?” (R5:2). Similar to the man quoted above, she likewise thought about how the radioactive material could travel: “If it spreads, I mean, in the air, if it goes into the groundwater and it goes around how damaged can you get?” (R5:2). The perception of the effectiveness of decontamination was also affected by perceptions of the impact of radiation over a long period of time. A young male asked, “At the moment, absolutely, it may be safe. But what does it really mean?” He pointed out that the impact can take place in the long term: “But then maybe you die in 20, 30 years because of it” (R9:2). A middle-aged man likewise pointed to a “low-intensity impact all the time” (R8:3), thus also doubting the effectiveness of remediation.

In addition to statements that expressed doubt about decontamination because the risk was perceived as being difficult to contain, respondents applied more holistic interpretations of what constitutes a successful recovery. As can be seen in Table 3, the respondents were not sure that radiation levels below the limit values would convince people to stay, which could affect the extent to which planned restoration takes place. A senior participant jokingly stated that it is “doubtful, with our life expectancy, if we [will] come back in. Because I mean, we are a little over 27 years old [laughs]” (R2:4). Further, younger people may decide that they are happy in their new place of residence and not want to uproot their family members once again. Without a sufficiently positive reaction to decontamination, the area could become depopulated and look like a “ghost town” (R7:3). Respondents indeed equated successful restoration with more than a reduced radiation dose; the area would also need to be restored visually, socially, and economically. A young man expressed that, “if you no longer have a garden, you may not want to live there, even if they say they have restored it” (R9:2). Again, restoration was perceived as a holistic project.

### 3.5. Disbelief in the Effectiveness of Remediation Is Associated with Preferences for More Frequent Dialogical Communication

**Figure 2:**
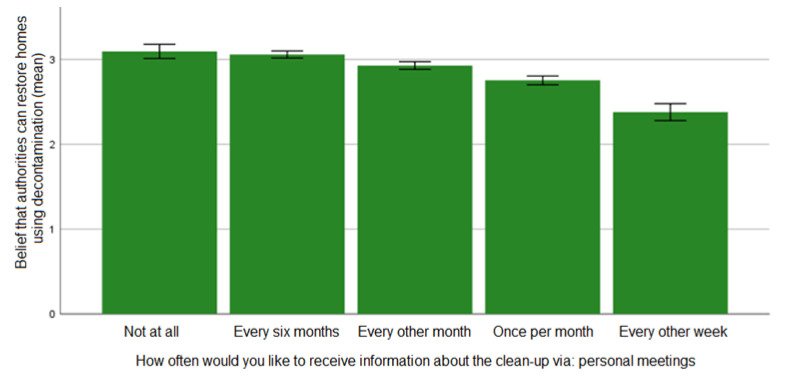
The relationship between respondents’ belief in the effectiveness of remediation and the need for more or less frequent personal meetings (error bars: ±1 SE).

In addition to worry being associated with preferences for more frequent communication activities with the possibility of dialogue, we found that lacking belief in the effectiveness of the remediation is associated with similar communication preferences. As shown in Figure 2, there is a monotonic relationship between the two variables: the less the respondents believe that the authorities can restore the residential area with the help of remediation, the more often they wish to be involved in dialogical communication. There were statistically significant differences between the following variable values: “every other week” and all other values (*p* < 0.05); “once per month” and all other values except for “every other month” (*p* < 0.05); and “not at all” + “once every six months” and all other values except for “every other month” (*p* < 0.05).

Furthermore, the more respondents doubted the effectiveness of remediation, the more common it was for them to express the need for in-person meetings with the authorities responsible for decontamination. We did not see the same effect on preferences regarding written information by letter or email. Those results show, if graphed, a high point in the middle and no statistically significant results (cf. Table A2, Appendix B).

### 3.6. Focus Group Discussions on Intentions to Either Accept or Avoid Potential Risk

When it comes to perceived dread risk, citizens prefer measures that remove the risk or separate people from it [46]. This was verified when the informants discussed whether they would stay in a decontaminated area (following specific rules of conduct) or move and start over somewhere else. The interview respondents who assessed dread risk saw, in practice, little opportunity to stay in the remediated area, even if the neighborhood and homes had been cleaned up to levels that are classified as safe and if authorities had informed and communicated with them about it. If respondents considered living in a remedied area, it was rarely because they preferred it; often, it was because circumstances such as low finances and housing unavailability would force them to do so. One of the respondents expressed their intention to move provided that adequate financial conditions existed: “I would move as far as I could and stay there as much as possible” (R5:1). A young woman articulated her intention to move in the following way: “I think that, if they would have said, ‘Move because there is a health risk’, I would have moved. Had they said that it was safe, then I would have moved anyway because I would not want to trust them” (R4:1). It seems unlikely that any particular mode or frequency of communication would change the intentions of citizens assessing dread risk. However, not everyone perceived the situation to involve dread risk. A female respondent in her 50s made an unusual statement: “I might be a little too fearless to actually think about risks and consequences and such and I have great confidence in our authorities, I really do” (R10:1). When discussing the scenario, she considered staying in the area with her husband. As in the section related to perspectives on the risk of radiation, the informants expressed care for children as impacting their views; it was stated as a reason to move from a decontaminated area (Table 4).

The same sentiments were applied to pets, whose movements can hardly be controlled if behavioral restrictions apply. Another element that makes it less attractive to stay is that a decontaminated area encompasses regulations regarding mobility, potentially indefinitely. A man in his 30s captured several of these aspects in the following statement: “I would not like to think about where I go, what I do, all the time. Even though I might feel safe in the house perhaps, a lot is going on all around. So, there is a lot to think about—family, children, dogs, animals. It’s probably easier to just move and forget about it.” (R8:1).

As shown in Table 4, the idea of having to think about risks and adapt one’s behavior for a long time to come—despite the fact that extensive decontamination has already been implemented—was not appealing to the interviewees. The authorities’ safety measures would ideally result in risk management coming to an end. Because it would likely be perpetuated—through both prolonged restrictions and radiation monitoring—respondents would consider moving from the area to avoid the further need for risk management and individual risk responsibility (i.e., allowing “normal life” to resume).

Finally, the discussions related to settlement featured many practical issues, mentioned in the points at the end of Table 4. Risks other than environmental hazards, such as a weak economy, poor community services, and housing only being available in crime-ridden areas, were mentioned as obstacles when taking a settlement decision.

### 3.7. Preferences for Leaving the Remediated Area Are Associated with Preferences for More Frequent Dialogical Communication

**Figure 3:**
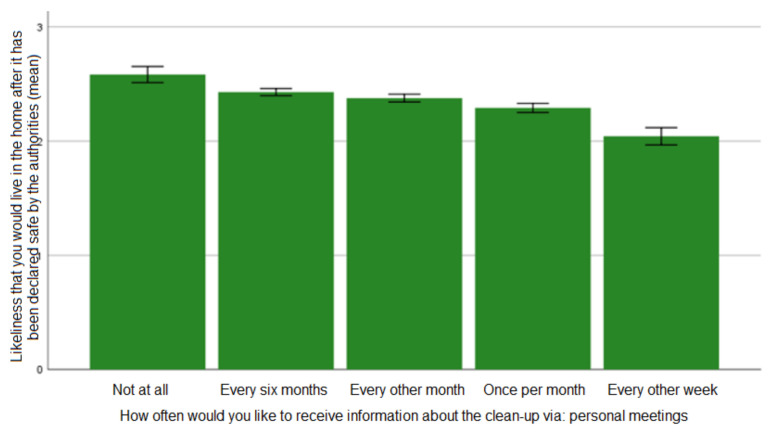
The association between preferences for risk acceptance and preferences for information about remediation through personal meetings (error bars: ±1 SE).

Another measurement of risk attitude—whether respondents found it probable that they would stay after decontamination—also showed a clear relationship with preferences regarding communication. As shown in Figure 3, the less likely the respondents found it that they would stay and continue living in the remediated area, the more often they wished to have the opportunity for communication through personal meetings during the remediation process. Statistically significant differences appeared between “every other week” and all other values (*p* < 0.05); “once per month” and all other values except for “every other month” and “once every six months” (*p* < 0.05); and “not at all” and all other values except for “once every six months” and “every other month” (*p* < 0.05).

Preferences regarding the frequency of written information, however, peak in the middle if graphed in a bar chart, and in fact the most risk avoidant alternative corresponds with the least need for written information (cf. Table A2, Appendix B).

## 4. Discussion and Conclusions

The extent of damage that NPP accidents can cause to public health and the environment continue to cause concern, necessitating further understanding of not only reactor safety but also citizens’ attitudes and behaviors that would affect safety measures in communities in the event of an accident [12]. Applying a mixed methods approach combining qualitative and quantitative data, focus groups were used to gain an in-depth understanding of citizens’ attitudes to radiation risk and their communication preferences in a remediation scenario. In addition, survey data was analyzed to gauge the association between key indicators of risk attitude and communication needs.

The focus group results showed the breadth of lay approaches to risk and risk communication, and the importance of a holistic perspective on remediation. Particularly uniquely, the results indicate that individuals’ concerns about children’s health are as multifaceted as are their relationships with children; they thus involve not only people’s concern for their own children but also for siblings and nieces (as per younger respondents), grandchildren (as per senior respondents), and more. Although other studies have shown parents’ concerns about children’s health in remediation situations, particularly mothers [25], all focus groups in our study actually voiced an altruistic attitude towards the youngest who may be at risk. This finding from our exploratory focus group study generates the hypothesis that in the event of a nuclear accident in a country with relatively high gender equality [52], with a less rigid division of labor between the sexes, a wider range of people may worry about children’s health and act in what they perceive as children’s best interests. Hence, a masculine, ‘macho’ attitude towards potential risk may not be so common either. Thereby, broader masses may direct criticism towards restoration projects that entail some health risk due to low-dose radiation exposure, which they can recognize quite concretely due to restrictions being in effect in recovery areas.

Another novel finding is that pets—which naturally want to sniff around during walks in green areas—were included in some respondents’ circles of concern in a remediated area with elevated radiation levels, adding incentive to move permanently. This is very likely also an issue that is more pronounced in our findings in a Western cultural setting, than would be the case in a region where it is unusual for a dog to be part of a household and for it to live indoors and be taken out for walks. In addition, the analysis of focus groups showed that citizens’ attitudes to radiation risk and the effectiveness of decontamination are rooted in the notion that the risk itself is difficult to control both temporally (i.e., with effects over a long period of time, possibly several generations) and spatially (i.e., that it can be preserved or transferred through groundwater, wind, etc.). Although this does not in any way imply new knowledge about radioactive material, it shows that laypeople assess the effectiveness of decontamination not only on the basis that radiation levels are below a limit value, but on perceptions of the challenging nature of the risk, and also on how life as a whole will be affected in the area in the long run. Regarding communication, all focus groups expressed the need for both swift information, and ahead of critical phases more reflective dialogue with those responsible. A stand-out result regarding communication was how strongly younger respondents preferred involvement through modern technology, both in terms of information on demand 24/7 and radiation measurement for residents staying in the decontaminated area.

Specifying certain results regarding communication, the analysis of survey data (n = 2291) and three indicators of risk attitude (i.e., degree of worry, remediation effectiveness, and intentions to stay) showed that the more negative an individual’s attitude is toward the remediation scenario, the more opportunities they want to be involved in communication that allows for dialogue. Thus, while research on the risk information seeking and processing (RISP) model has shown that more frequent one-way information is needed when citizens harbor negative risk attitudes [39], the present study adds evidence that, in cases of perceived dread risk, levels of worry and preferences for risk avoidance do not seem to affect preferences regarding the frequency of information very much. What sets the more concerned and risk-averse citizens apart seems to be their need for more frequent dialogical communication with those responsible for risk management. The fact that this group also turned out to be large, comprising just over half of the respondents, only makes the results more important to consider for decision-makers and risk managers.

In connection to this, intentions to tolerate risk and return after evacuation appeared to relate to things other than the quality of the information and communication provided. The decision to stay is mainly determined by risk perception, general stigmatization of the residential area, belief in the effectiveness of decontamination, relatives choosing to stay, and/or school or job opportunities in the affected area. The importance of social ties, for example, was very important to the respondents, as has been confirmed by other studies [41]. However, the respondents also perceived some important obstacles to permanently moving away from a decontaminated area, including great individual and/or household financial loss. For those who would choose to return to the decontaminated area, the interviews demonstrated that citizens’ involvement in the remediation process is highly valued. Not only personal meetings were mentioned in the interviews but also broader involvement through access to radiation measurements and information on the basis of need.

Moreover, according to the focus group data, citizens associate a recovery scenario necessitating evacuation with serious accidents such as those in Chernobyl and Fukushima, triggering associations with their own mediated experiences and stories of dread risk. Commonly, citizens imagine that future accidents and recovery attempts will generate strong risk aversion, the propensity to flee, conflicting values, victims facing injustice, and jeopardized trust in the institutions responsible for the whole process. The life situation in a partially polluted area (i.e., following remediation efforts) is also considered by groups of citizens to create life-threatening and difficult-to-handle dilemmas. It is obvious, then, that the conditions for information and communication work between authorities and victims will become very complex and difficult. The results therefore also suggest that the great confidence shown both in previous studies [29] and in guidelines on nuclear emergency management [6] in the effects of correct and coordinated information may be too optimistic.

The lessons to be drawn for policy and practice from the study include that citizens have very high demands on a decontaminated area—with everything from very low radiation dose levels, to job opportunities, and permission for various outdoor activities—if the alternative of moving should not seem more reasonable. This is a challenging issue to resolve, as extensive remediation is difficult and expensive to implement. Furthermore, responsible agencies should prepare for both rapid information provision, but also in-person meetings with residents that allow for the exchange of thoughts and ideas. At such meetings, however, our results indicate that responsible actors should expect an over-representation of critically minded stakeholders who do not share the authorities’ overall goal that citizens will move back after evacuation. Still, to openly engage in such conversations, listening and answering questions, can be reassuring and build important trust. In addition, the prospects for successful communication for all involved would be better if policies allowed a higher degree of freedom of action for those affected—for instance, through financial compensation and not just a plan to relocate everyone. Otherwise, the responsible state agencies will likely face an uphill battle as they attempt to convince large groups of citizens that they should tolerate a possible risk that they will not, in reality, tolerate. Previous major accidents also show that planned restoration and actual results differ greatly, as has been the case in several areas of the Fukushima prefecture [15,18].

Finally, it should be pointed out that our study is subject to certain limitations. Even though we have combined qualitative (i.e., interview) and quantitative (i.e., survey) data in a mixed methods approach, all our results specifically concern the Swedish context. Based on cultural and policy similarities, the results are relevant for other countries as well; however, more countries need to be studied in a similar way to increase our knowledge of the effects of different national circumstances in the event of a nuclear accident involving evacuation, decontamination, and relocation. Furthermore, the present study examined attitudes to a hypothetical scenario, and respondents’ behavioral intentions indicate preferences rather than actual actions constrained by life circumstances. Without, for example, financial compensation, more people than indicated by our study may choose to stay in a decontaminated area, for reasons such as having mortgages while the value of their properties plummet. Future studies can highlight any potential hypothetical bias by more systematically comparing scenario-based studies and research on accident-affected areas.

## Figures and Tables

**Table 1:** A summary of focus group findings on perceptions of ionizing radiation and health risks.

Perceived Transboundary Risk Source	Perceived Health Risks
- Potential adverse effects for future generations were suggested. - There was a perceived risk of internal and genetic impact. - There was a perceived residual risk to the environment. - Yet some expressed that radiation levels subside over time. - It was suggested that radioactive material can be passed on through the weather. - Some suggested that radioactive material can be passed over areas by wild animals and transported food. - Some suggested that radioactive material can be carried indoors, on one’s shoes or by one’s dog.	- Judgements of risk to health varied widely. - On one hand, low-dose radiation was described as a low risk to health. - On the other hand, low-dose radiation was described as uncertain and possibly life-threatening (i.e., a matter of “life and death”), carcinogenic, and having adverse effects on reproduction (e.g., sterility and fetus morbidity). - Respondents expressed notions that children are particularly vulnerable to radiation. - The health of children was perceived as particularly necessary to protect and as a crucial responsibility. - The risk to dogs and other family animals, whose behavior seems difficult to control in case of behavioral restrictions, was also mentioned.

**Table 2:** A summary of focus group findings on information and communication preferences in the remediation phase.

Preferences Regarding the Frequency of Information	Preferences Regarding Participatory Communication
- When making decisions or changes, rapid and frequent information was preferred. - During normal work, frequency preferences varied from daily to biweekly. - To accommodate preference variation, informants requested frequent information digitally (e.g., through an application). - General information was desired more frequently early on (e.g., in the first year) than in later phases.	- As soon as possible and when decisions regarding the neighborhood would affect them, informants preferred face-to-face interaction. - If they still had a home in the area, informants preferred solutions that would allow participatory processes, such as access to radiation measurement tools and results.

**Table 3:** A summary of focus group findings on challenges associated with decontamination.

Radioactive Material Is Difficult to Contain	Remediation Involves Unusual or Unknown Measures	Decontamination Does Not Guarantee Holistic Community Restoration
- Radioactive material is perceived as difficult to contain spatially. - Radioactive material is perceived as difficult to contain across time. - Clean-up personnel may be exposed and affected. - Others may be accidentally exposed despite remediation efforts.	- Authorities’ inexperience with NPP accidents and remediation will likely reduce clean-up effectiveness. - The public’s lack of knowledge about remediation makes the effectiveness of remediation a matter of trust. - Respondents expressed that they prefer stronger guarantees than relying on trust.	- People may feel comfortable in the area to which they have evacuated. - Older citizens may not have the time or energy to return. - Completed decontamination does not put an end to risk management for the individual. - Despite successful remediation, house prices are likely to plummet. - Despite successful remediation, the area could become a “ghost town”.

**Table 4:** A summary of focus group findings on reasons to move from, or stay living in, a decontaminated area.

Reasons to Move from a Decontaminated Area	Reasons to Stay in a Decontaminated Area
- Perceived dread risk (severe and transboundary). - Children and/or outdoor animals belong to the household. - Dissatisfaction with restrictions on outdoor activities. - Measures should end, not perpetuate, risk management. - Moving puts an end to the worry over risk. - People move because of less significant events than a nuclear accident. - General stigmatization. - Relatives who choose differently and do not visit. - Fewer job opportunities. - Less access to care, communications, and services.	- Perceived tolerated risk. - Only adults belong to household, and if domestic animals do, they are content indoors. - The home represents highly valued security. - The hometown offers strong social ties. - Work is strongly associated with the area. - Available housing is too far away. - Available housing involves other serious risks, such as crime. - The move means too great a financial loss, especially if compensation is insufficient.

## Data Availability

Third party survey data. Restrictions apply on the availability of these data. Data was obtained from The Laboratory of Opinion Research (LORE) and are available from the lead author with the permission of LORE.

## Appendix A

A nuclear accident has occurred in Sweden, and your residential area is contaminated with radioactive substances. You are evacuated to temporary housing while the authorities are cleaning up parts of your residential area. The remediation includes the removal of contaminated land close to your house, as well as the cleaning and removal of radioactive material from your house’s roof, facade surfaces, and, if necessary, indoor surfaces. The disposal of adjacent land is likely to damage vegetation such as flowerbeds. The remediation measures may take up to one year to complete.

After the clean-up, measurements of houses and gardens show that the levels of radioactive substances are so low that they are considered harmless. However, there are areas around your residential area that show levels of radiation that are so high that you are not allowed to live there, and in some cases, they require special permits for access. The authorities advise parents not to allow their children to play freely in surrounding natural areas. They also advise against hunting and picking berries and mushrooms. Some industries (especially hunting, fishing, and agriculture) may find that selling certain products is prohibited or difficult. The following questions attempt to determine how you relate to living in such a residential area.

## Appendix B

**Table A1:** An overview of the association between attitudes to the remediated area (three survey items) and frequency preferences regarding in-person meetings.

Frequency Preferences for in-Person Meetings with the Agency Responsible for Decontamination	Worry Over Ionizing Radiation (Mean)	N	Std. Deviation
Not at all	3.07	201	1.313
Once every six months	3.34	760	1.220
Every other month	3.43	553	1.126
Once a month	3.67	430	1.111
Biweekly	4.07	145	1.032
Total	3.46	2089	1.193
**Frequency Preferences for in-Person Meetings with the Agency Responsible for Decontamination**	**Belief in Decontamination Effectiveness (Mean)**	**N**	**Std. Deviation**
Not at all	3.09	201	1.177
Once every six months	3.06	758	1.139
Every other month	2.93	552	1.055
Once a month	2.75	430	1.073
Biweekly	2.38	145	1.202
Total	2.92	2086	1.127
**Frequency Preferences for in-Person Meetings with the Agency Responsible for Decontamination**	**Preference to Live in a Remediated Area (Mean)**	**N**	**Std. Deviation**
Not at all	2.58	201	1.002
Once every six months	2.43	759	0.861
Every other month	2.38	551	0.806
Once a month	2.29	428	0.830
Biweekly	2.04	145	0.904
Total	2.37	2084	0.866

**Table A2:** An overview of the association between attitudes to the remediated area (three survey items) and frequency preferences regarding written information.

Frequency Preferences for Receiving Information about the Decontamination via E-Mail or Letter	Worry Over Ionizing Radiation (Mean)	N	Std. Deviation
Not at all	3.37	41	1.356
Once every six months	3.27	37	1.367
Every other month	3.15	159	1.244
Once a month	3.31	854	1.170
Biweekly	3.62	1037	1.179
Total	3.45	2128	1.199
**Frequency Preferences for Receiving Information about the Decontamination via E-mail or Letter**	**Belief in Decontamination Effectiveness (Mean)**	**N**	**Std. Deviation**
Not at all	2.56	41	1.343
Once every six months	2.78	37	1.294
Every other month	3.09	158	1.127
Once a month	2.96	852	1.125
Biweekly	2.89	1035	1.114
Total	2.92	2123	1.129
**Frequency Preferences for Receiving Information about the Decontamination via E-mail or Letter**	**Preference to Live in a Remediated Area (Mean)**	**N**	**Std. Deviation**
Not at all	2.29	41	0.929
Once every six months	2.27	37	0.962
Every other month	2.62	159	0.863
Once a month	2.43	852	0.858
Biweekly	2.32	1034	0.864
Total	2.38	2123	0.868
